# Fractionated Radiotherapy with 3 x 8 Gy Induces Systemic Anti-Tumour Responses and Abscopal Tumour Inhibition without Modulating the Humoral Anti-Tumour Response

**DOI:** 10.1371/journal.pone.0159515

**Published:** 2016-07-18

**Authors:** Thomas H. P. M. Habets, Tammy Oth, Ans W. Houben, Mirelle J. A. J. Huijskens, Birgit L. M. G. Senden-Gijsbers, Melanie C. A. Schnijderberg, Boudewijn Brans, Ludwig J. Dubois, Philippe Lambin, Marijke De Saint-Hubert, Wilfred T. V. Germeraad, Marcel G. J. Tilanus, Felix M. Mottaghy, Gerard M. J. Bos, Joris Vanderlocht

**Affiliations:** 1 Division of Hematology, Department of Internal Medicine, GROW—School for Oncology and Developmental Biology, Maastricht University Medical Center +, Maastricht, The Netherlands; 2 Tissue Typing Laboratory, Department of Transplantation Immunology, GROW—School for Oncology and Developmental Biology, Maastricht University Medical Center +, Maastricht, The Netherlands; 3 Division of Nuclear Medicine, Department of Internal Medicine, School of NUTRIM, Maastricht University Medical Center +, Maastricht, The Netherlands; 4 MaastRO Laboratory, Department of Radiation Oncology, GROW—School for Oncology and Developmental Biology, Maastricht University Medical Center +, Maastricht, The Netherlands; 5 CiMaas BV, Maastricht, The Netherlands; Technische Universitaet Muenchen, GERMANY

## Abstract

Accumulating evidence indicates that fractionated radiotherapy (RT) can result in distant non-irradiated (abscopal) tumour regression. Although preclinical studies indicate the importance of T cells in this infrequent phenomenon, these studies do not preclude that other immune mechanisms exhibit an addition role in the abscopal effect. We therefore addressed the question whether in addition to T cell mediated responses also humoral anti-tumour responses are modulated after fractionated RT and whether systemic dendritic cell (DC) stimulation can enhance tumour-specific antibody production. We selected the 67NR mammary carcinoma model since this tumour showed spontaneous antibody production in all tumour-bearing mice. Fractionated RT to the primary tumour was associated with a survival benefit and a delayed growth of a non-irradiated (contralateral) secondary tumour. Notably, fractionated RT did not affect anti-tumour antibody titers and the composition of the immunoglobulin (Ig) isotypes. Likewise, we demonstrated that treatment of tumour-bearing Balb/C mice with DC stimulating growth factor Flt3-L did neither modulate the magnitude nor the composition of the humoral immune response. Finally, we evaluated the immune infiltrate and Ig isotype content of the tumour tissue using flow cytometry and found no differences between treatment groups that were indicative for local antibody production. In conclusion, we demonstrate that the 67NR mammary carcinoma in Balb/C mice is associated with a pre-existing antibody response. And, we show that in tumour-bearing Balb/C mice with abscopal tumour regression such pre-existing antibody responses are not altered upon fractionated RT and/or DC stimulation with Flt3-L. Our research indicates that evaluating the humoral immune response in the setting of abscopal tumour regression is not invariably associated with therapeutic effects.

## Introduction

The main goal of optimized radiotherapy (RT) is to maximize the therapeutic ratio in which tumours receive a high dose of irradiation while sparing the normal healthy tissue. However certain radiation-induced bystander effects of non-irradiated cells exhibit biological effects which can be beneficial for treatment outcome [1–3]. Already in 1953, R.J. Mole described the ‘abscopal’ effect (*ab*: *position away from; and scopos*: *a target for shooting at*), in which tumour cells regressed or even disappeared outside the field of primary irradiation [4, 5]. Although this phenomenon is certainly infrequent it has become clear that it is not confined to a specific type of cancer. So far, several clinical case studies reported the regression of non-irradiated metastasis after conventional RT combined with or without immunotherapy [6, 7]. These abscopal effects were observed in melanoma [8–12], lung carcinoma [13, 14], renal cell carcinoma [15], hepatocellular carcinoma [16], and chronic lymphocytic leukemia [17]. Until now, the complete picture of the biologic mechanisms underlying these radiation-induced bystander effects remains elusive, but the most likely explanation is that non-irradiated tumour regression is a consequence of systemic immune activation induced by immunogenic cell death (ICD) of irradiated tumour tissue [10, 18].

Over the last decades significant progress was made in our knowledge on how the immune system can be activated by injured tissue and various molecular pathways triggered by damage-associated molecular patterns (DAMPs) [19]. Therefore, authors postulated that abscopal tumours regress as a result of ICD by irradiated tumours that in turn activate both the local and systemic immune system [20]. As a consequence of this irradiation, dying tumour cells can release DAMPs that activate dendritic cells (DC) [19, 21] allowing them to travel to adjacent lymph nodes, present their tumour-associated antigenic material in the context of major histocompatibility complex (MHC) class I and class II molecules and induce both antigen-specific T- and B cell responses [18, 22]. Due to systemic release of these antigen-specific cells and their secreted products both the primary irradiated and non-irradiated tumour cells are eradicated. Even though this phenomenon is only occasionally observed in patients that are treated with RT only, it indicates that the immune system harbours potent pathways for the control of metastatic tumours and the identification of these pathways may reveal targets for therapeutic intervention.

In an effort to gain fundamental insight into the immunological mechanism of the abscopal effect, Demaria *et al*. described an animal model (67NR mammary carcinoma) in which RT also influenced the tumour outside the field of irradiation [23]. In this model they further showed that this inhibition of the secondary tumour is not observed if an unrelated tumour (the murine B cell lymphoma A20) was inoculated as a secondary tumour indicating that the anti-tumour effect was antigen-specific. Importantly, they demonstrated that these effects were not observed in immunodeficient (*nude)* mice, indicating that T cells were necessary for radiation-induced bystander effects [23]. These authors confirmed the importance of T cells by combining fractionated RT and CTLA-4 blockade that resulted in CD8 T cell-mediated tumour regression of irradiated and distant non-irradiated tumours [24].

In line with observations on the importance of T cells, also evidence was provided that stimulating the endogenous DC compartment augments the efficacy of the RT-induced activity against metastasis [23–25]. Treatment with FMS-like tyrosine kinase 3 ligand (Flt3-L) to stimulate DC and local RT in a mouse model for Lewis carcinoma showed a reduction of metastases and prolonged disease-free survival [25]. This was also observed by combining RT and Flt3-L administration that resulted in tumour regression of both irradiated and distant non-irradiated tumours [23]. Furthermore, another essential DC growth factor granulocyte-macrophage colony stimulating factor (GM-CSF) stimulated a robust and long-lived anti-tumour immune response in a murine melanoma model [26]. Importantly, Golden *et al*. recently showed that the concept of DC stimulation extends beyond animal models and they provided proof-of-principle that stimulating the endogenous DC compartment with GM-CSF combined with RT resulted in objective abscopal responses in more than 20% of patients with metastatic cancer. The presence of abscopal effects was also associated with a survival benefit in these patients [27].

Taken together these findings clearly indicate the importance of DC-induced T cell responses in abscopal tumour regression. This concept is further supported by the fact that abscopal effects can be enhanced by immune checkpoint inhibitors such as CTLA-4 blockade in animal models [24] as well as in clinical studies [10]. However, these studies do not preclude that other immune mechanisms play additional roles in radiation-induced bystander effects. Although the study design of Golden *et al*. was not aimed for immune monitoring, they showed differences in baseline blood parameters in abscopal responders and non-responders [27]. Additionally, Postow *et al*. observed that the abscopal effect was paralleled with the induction of antibody responses against the tumour in a patient with metastatic melanoma [10]. In this patient a pre-existing antibody response to the melanoma antigen NY-ESO-1 was boosted 30 fold upon treatment correlating with the time of disease resolution. Besides boosting of the pre-existing B cell response, the patient also displayed antibodies to a different epitope of the same antigen. Furthermore, seromic analysis of the patient’s serum showed an induction of antibody responses against ten novel cancer antigens. A similar effect was observed in a different study in which a melanoma patient displayed an increased level of MAGE-3 antibodies upon radiation-induced abscopal effects [11].

Nowadays, it remains to be established whether the induction of antibody responses against the tumour is directly involved in abscopal tumour inhibition, or whether the induction of such antibodies only serves as a biomarker indicating the development of novel immune responses in a subset of patients. Alternatively, it is possible that the presence of such antibody responses is a prerequisite for abscopal effects.

In the current study, we aimed to assess whether the abscopal tumour growth delay that is observed after fractionated RT is paralleled with the modulation of a pre-existing humoral anti-tumour response in the 67NR mammary carcinoma animal model. In addition, we assessed whether stimulation of plasmacytoid (pDC) and conventional (cDC) DC with Flt3-L alone or combined with fractionated RT can modulate a pre-existing humoral immune response.

## Materials and Methods

### Cell culture

The murine (Balb/C) 67NR mammary carcinoma cell line was kindly provided by S. Demaria (Department of Pathology, New York University School of Medicine, New York, New York). The 67NR cell line was cultured in Dulbecco’s Modified Eagle Medium (DMEM 1X with 4.5g/L D-glucose, L-glutamine, and pyruvate; Gibco) supplemented with 10% fetal calf serum (Greiner Bio One) and 1% penicillin-streptomycin (Gibco). The metastatic murine 4T1 mammary carcinoma (a differentiated subclone of the non-metastatic 67NR cell line) and C26 colon carcinoma cell lines (both obtained from the ATCC) were cultured in Roswell Park Memorial Institute Medium (RPMI 1640 1X with L-glutamine, and 25mM HEPES; Gibco) supplemented with 10% fetal calf serum and 1% penicillin-streptomycin. The cell lines were incubated in a humidified 20% O_2_ and 5% CO_2_ chamber at 37°C.

### Mouse model

In order to investigate abscopal tumour regression, we took advantage of the mouse model previously generated and optimized by Demaria *et al*. [23]. In short, a total of 1 *10^5^ 67NR cells were subcutaneously injected in immunocompetent Balb/C mice (8 to 10 weeks of age; Harlan Laboratories). The mice were anesthetized with isoflurane (IsoFlo, Abbott) prior to cell injections. The cells were injected in the right (primary tumour) and left flank (secondary tumour) at day 0 and day 2 respectively. At day 14, tumour-bearing mice were randomised based on primary tumour size and assigned to four groups: untreated, Flt3-L, RT, RT+Flt3-L. The tumour-bearing mice were housed separately, had free access to food and water, and were checked every day for animal well-being according to local institutional guidelines for animal welfare. The tumour size was assessed three times a week by palpation (caliper; three dimensions), and corrected using the following formula: L*W*H*(π/6) [28]. At the predefined human endpoint (maximum volume of 2cm^3^ for both tumours) or at the end of the experiment (day 162), mice were anesthetized and sacrificed by cervical dislocation. None of the mice died without euthanasia. The blood and both tumours were collected for further analysis. The blood was incubated with heparin (LEO Pharma) and centrifuged to separate the plasma. Half of the tumour was homogenized and used as a single cell suspension for flow cytometric analysis and the other half of the tumour was embedded in TissueTec and further stored at -80°C for immunohistochemistry (IHC). The animal experiments were performed according to Dutch government guidelines, approved by the Animal Ethical Committee of Maastricht University (Maastricht, The Netherlands, permit number DEC2010-171).

### Treatments

#### Fractionated radiotherapy (RT)

**Fig 1:**
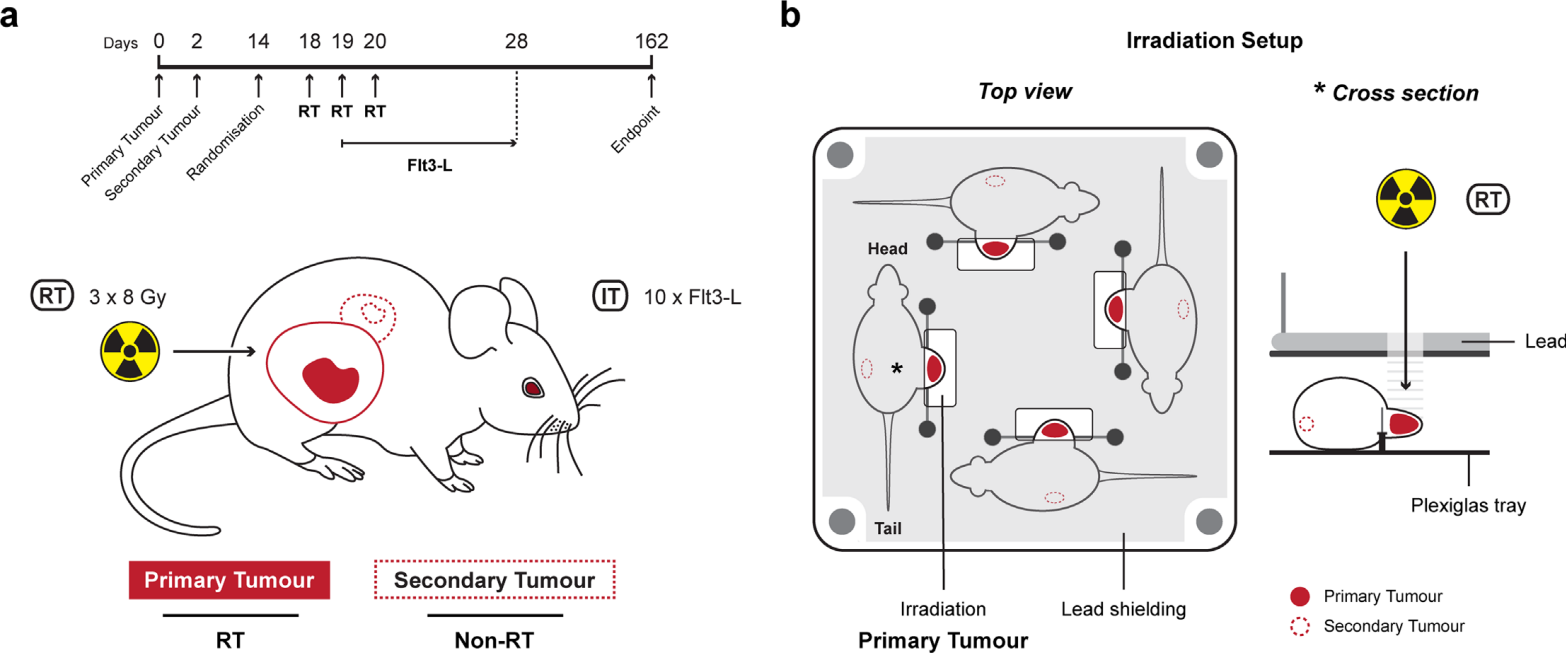
Mouse model to investigate the abscopal effect. a. Schematic representation of the 67NR mammary carcinoma Balb/C mouse model and the timeline of treatments. 1 *10^5^ 67NR tumour cells were inoculated in the right (primary, straight line) and left (secondary, dashed line) flank on day 0 and 2, respectively. At day 14, animals were randomised based on the primary tumour size and assigned to four groups (untreated, Flt3-L, RT, RT+Flt3-L). Fractionated radiotherapy (RT, 3 x 8 Gy) was only given to the primary tumour at day 18, 19, and 20. Immunotherapy (IT, DC growth factor Flt3-L) was administered for ten consecutive days, started at day 19. The endpoint was at day 162 or a total tumour volume of 2cm^3^. b. Schematic representation of the irradiation setup. Four tumour-bearing mice (per session) were positioned in a customized Plexiglas tray. The animals’ body was protected by lead shielding except for the primary tumour that was placed under a linear accelerator. The star (*) in the top view indicates the position of the cross section view.

Only the primary tumour was irradiated with three times 8 Gy (±45.5 mGy/s) in three consecutive days (Fig 1A). The tumour-bearing mice in groups RT and RT+Flt3-L were anesthetized via *i*.*p*. injection of pentobarbital (60mg/mL in NaCl 0.9%). The mice (four per session) were positioned in a customized Plexiglas tray. The animals’ body was protected by lead shielding except for the primary tumour that was placed under a linear accelerator (Varian Truebeam; 15 MeV electrons) as is schematically illustrated in Fig 1B.

#### Growth factor

The tumour-bearing mice in groups Flt3-L and RT+Flt3-L received *i*.*p*. injections of 10μg Flt3-L (human Flt3-L; Miltenyi Biotec) for ten consecutive days as previously described [23]. The administration of Flt3-L started at day two of RT (Fig 1A).

### Vaccination of mice with heat-killed tumour cells

In order to obtain heat-killed 67NR cells, a total of 10 *10^6^ 67NR cells were cultured, harvested in PBS, and heat-killed for 30min at 60°C. The heat-killed cells were resuspended in incomplete Freund’s adjuvant (IFA; Sigma) containing 200μg DNAse, and 0.5mg/mL CpG ODN 1826 (VacciGrade, InvivoGen). The mixture was injected in immunocompetent Balb/C mice. Primary immunized mice received a booster injection containing IFA, CpG ODNs and heat-killed 67NR cells after 3 weeks. The immunized mice were anesthetized and sacrificed by cervical dislocation 1 to 3 weeks after the boost injection. The blood was collected by cardiac puncture and centrifuged to separate the plasma.

### Cellular flow cytometry and ELISA-based systems for the detection of tumour-binding antibodies (TBAs)

We optimized two independent methods for demonstrating the presence of TBAs in the plasma of 67NR tumour-bearing mice. For both methods we intentionally used different antibody clones for detection. In this manner, we used fluorescent detection with flow cytometry (FC), which has the advantage of a higher dynamic range in samples with a higher concentration. The second method used enzymatic amplification for a more sensitive detection in the lower concentration range.

#### Flow cytometry

1 *10^5^ 67NR cells were transferred to a 96 wells v-bottom plate. The cells were incubated with 10% FCS for 30min (Greiner Bio One) to prevent non-specific binding. Then, diluted plasma (1:20) samples were added for 30min at room temperature (RT). TBAs in the plasma were stained with APC labelled anti-mouse pan-Ig (polyclonal; BD) for 20min (RT). In case of immunoglobulin (Ig) isotype determination in the plasma, APC labelled anti-mouse IgG_1_ (clone RMG1-1, BioLegend), PE labelled anti-mouse IgG_2a_ (clone RMG2a-62, BioLegend), FITC labelled anti-mouse IgG_2b_ (clone RMG2b-1, BioLegend), and PE-Cy7 anti-mouse IgM (clone RMM-1, BioLegend) were used. In between all steps, cells were washed with buffer (PBS 1X, 1% FCS, 0.02% NaN_3_) and centrifuged for 3min, 800g at 4°C. The stained samples were measured and analysed using the FACSCanto II (BD) and FACSDiva software v6.0 (BD) respectively.

#### ELISA

1 *10^5^ 67NR cells were added to a flat-bottom 96 wells cell culture plate and cultured for 18hrs at 37°C. The adherent cells were washed with buffer (PBS 1X, 0.05% Tween 20) and fixated with 3.8% formaldehyde (Sigma) for 45min at RT. To prevent non-specific binding, the plate was subsequently incubated with 1% BSA (Sigma) for 1h at RT. Then, diluted plasma (1:20) samples of the intervention mice were added for 1h at RT. TBAs in the plasma were detected with HRP labelled anti-mouse pan-Ig (polyclonal; Dako) and TMB substrate (Thermo Scientific). The enzymatic reaction was stopped by 0.9M H_2_SO_4_. In between all steps, 67NR cells were washed with buffer. The absorbance was measured at 450nm (BioTek Synergy HTX).

### Cytometric bead assay for the determination of Ig isotypes in tumour lysates

The immunoglobulin isotypes that were present in the tumour tissue were determined using the cytometric bead array (CBA) kit (BD). TissueTec embedded tumour tissue was homogenized using a mini-bead beater (BioSpec) for 2x20s and 1x30s in CellLytic MT Mammalian Tissue Extraction buffer (Sigma). The total protein concentration in μg/mL was measured using the BCA (bicinchoninic acid assay) method (Thermo Scientific). The tumour lysates were incubated with microbeads coated with capture antibodies for the different Ig isotypes. The bound Ig isotypes were stained with fluorescent-labelled detection antibodies. Subsequently, the samples were measured using flow cytometry according to the manufacturers protocol. FCAP Array analysis software v3.0 (BD) was used for data analysis. The concentration of all Ig isotypes was normalized for total protein quantity of the tumour lysates.

### Detection of B cells and T cells by FC and IHC

#### Flow cytometry

1.5 *10^6^ tumour cells were added to a 96 wells v-bottom plate. The cells were incubated with mouse serum (Dako) to prevent non-specific binding (20min). Upon incubation, APC labelled anti-mouse CD45 (clone 30-F11), PE-Cy7 labelled anti-mouse CD11c (clone HL3), V500 labelled anti-mouse CD4 (clone RM4-5), FITC labelled anti-mouse CD19 (clone 1D3), V450 labelled anti-mouse CD8α (clone 53–6.7; eBioscience) were added for 15min at RT. The life-death marker 7-AAD (BD) was added before measurement. All fluorescent labelled antibodies were purchased from BD, unless otherwise specified. In between all steps, the cells were washed with buffer (PBS 1X, 1% FCS, 0.02% NaN_3_) and centrifuged for 3min, 800g at 4°C. The stained cells were measured and analysed (FACSCanto II and FACSDiva software; BD).

#### Immunohistochemistry

Half of the tumours embedded in TissueTec were sectioned (10μm) using a cryostat (Leica) and fixated with acetone. The unconjugated rat anti-mouse B220-CD45r, CD3, CD4, and CD8 antibodies (hybridoma cell lines) were added for 1h at RT. Then, peroxidase blocker and biotinylated anti-rat Ig (polyclonal; BD) were added for 30min at RT. These biotinylated antibodies were detected with HRP labelled streptavidin (30min) and DAB substrate (BD). The tumour sections were dehydrated in 50%, 70%, 96%, and 100% ethanol (5min) and analysed using the Carl Zeiss Axioskop 40 microscope.

### Figures and statistics

The graphs, figures, and statistical analyses were made with GraphPad Prism Pro version 6 (GraphPad Software) and combined in Adobe Illustrator CS6/CC (Adobe Systems). The Kaplan-Meier survival curves were compared using the log-rank (Mantel-Cox) test. The Mann-Whitney t-test was used to determine statistical significance between the treatment groups. For all data, a P value of < 0.05 (*) was considered to be statistically significant. P-values < 0.01 were graphically presented as (**).

## Results

### Fractionated radiotherapy (but not systemic DC stimulation with Flt3-L) of the primary tumour associates with delayed tumour growth, which translates into a survival benefit

In an effort to gain fundamental insight into the immunological mechanisms of the abscopal effect, we took advantage of the animal model that was developed and optimized by Demaria *et al*., in which RT of the primary tumour resulted in growth delay of the distant tumour outside the field of irradiation [23]. Furthermore, we wanted to address whether besides fractionated RT, stimulation of DC with growth factor Flt3-L would enhance the anti-tumour immune response in that animal model.

In this model, Balb/C mice were inoculated with a primary and secondary tumour by injecting 10^5^ 67NR tumour cells in both flanks with a temporal separation of 2 days between the primary (right flank) and the secondary (left flank) (Fig 1A). At day 14, we observed that in many animals the tumour growth differs in time of onset and in growth kinetics, therefore we randomised the animals based on their primary tumour size and assigned them to four groups (untreated, Flt3-L, RT, and RT+Flt3-L). Only the primary tumour received fractionated RT (8 Gy at day 18, 19, and 20), while the secondary tumour was non-irradiated (Fig 1B). Immunotherapy (IT) in terms of Flt3-L was administered for ten consecutive days (started at day 19).

**Fig 2:**
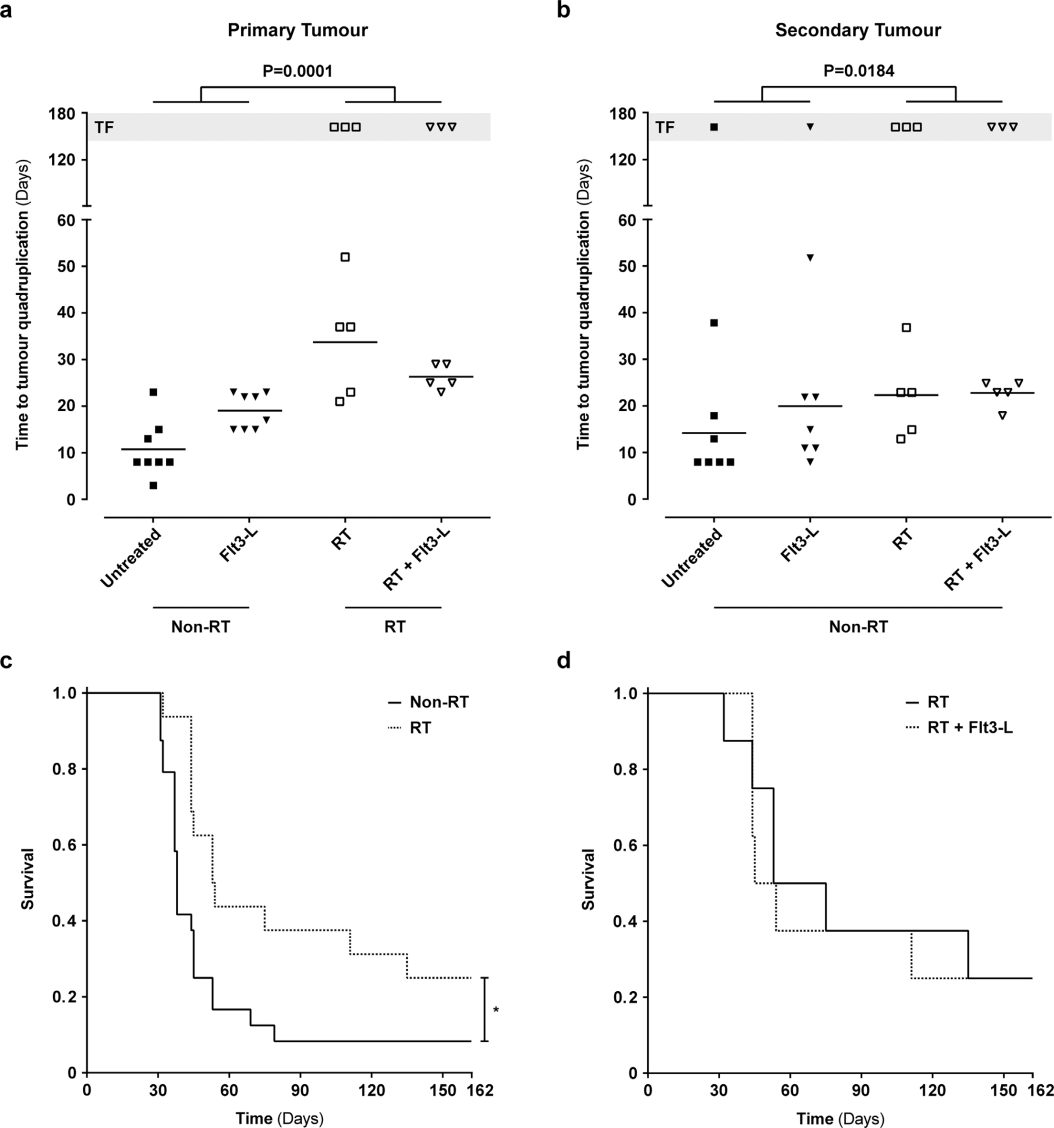
67NR tumour growth and survival following treatment with RT and Flt3-L in tumour-bearing mice. a, b. Time (days) to reach quadruplication of the primary and secondary tumour as measure for 67NR tumour growth. The measurement started 14 days after inoculation of the 67NR tumours. The primary and secondary tumour of the RT groups showed a growth delay as compared to the non-RT groups (P<0.05 *) (TF: tumour free mice are excluded from Mann-Whitney t-test). c, d. The Kaplan-Meier survival curves of the (un)-treated animals. The non-irradiated (non-RT) animals were untreated or received injections of Flt3-L (10 days). The irradiated (RT) animals received RT (3 x 8Gy) or RT+Flt3-L (3 x 8Gy + 10 days). Log-rank (Mantel-Cox) test P<0.05 *.

To evaluate tumour growth delay in mice that were non-irradiated or irradiated, we took the tumour growth variation into account and we decided to utilize the number of days to reach four times (quadruplication) the tumour volume that they had after randomization (day 14). The groups RT and RT+Flt3-L (RT) demonstrated a delay in primary (30 ±10 days) and secondary (23 ±7 days) tumour growth as compared to the groups untreated and Flt3-L (non-RT; primary 15 ±7 days; secondary 17 ±13 days) (Fig 2A and 2B; P<0.05). Furthermore, in the RT groups, there were six mice without a detectable primary tumour, as compared to zero mice in the non-RT groups (Fig 2A; TF). Additionally, we also observed six animals in the RT groups without a secondary tumour as compared to only two mice in the non-RT groups (Fig 2B; TF). Although the effect on the secondary tumour was not a uniform trend observed in all animals, the presence of more animals without a detectable secondary tumour in the RT groups was apparent (Fig 2B; TF). Importantly, the tumours that showed total regression (TF) at the end of the experiment was not a feature confined to animals with a small tumour at the point of randomization (primary 8.3 ±4.5 mm^3^; secondary 3.9 ±4.5 mm^3^) (S1 Fig).

To determine whether fractionated RT and DC stimulation with Flt3-L is associated with a survival benefit, we followed the untreated and treated animals for 162 days. As shown in Fig 2C, there was a significant survival benefit in mice treated with RT (fraction survival 0.25) as compared to mice that did not receive RT (fraction survival 0.08; P<0.05). However, we could not observe a survival benefit with Flt3-L alone (data not shown), nor in combination with RT (Fig 2D).

### Detection of TBAs in the plasma of 67NR tumour-bearing Balb/C mice

**Fig 3:**
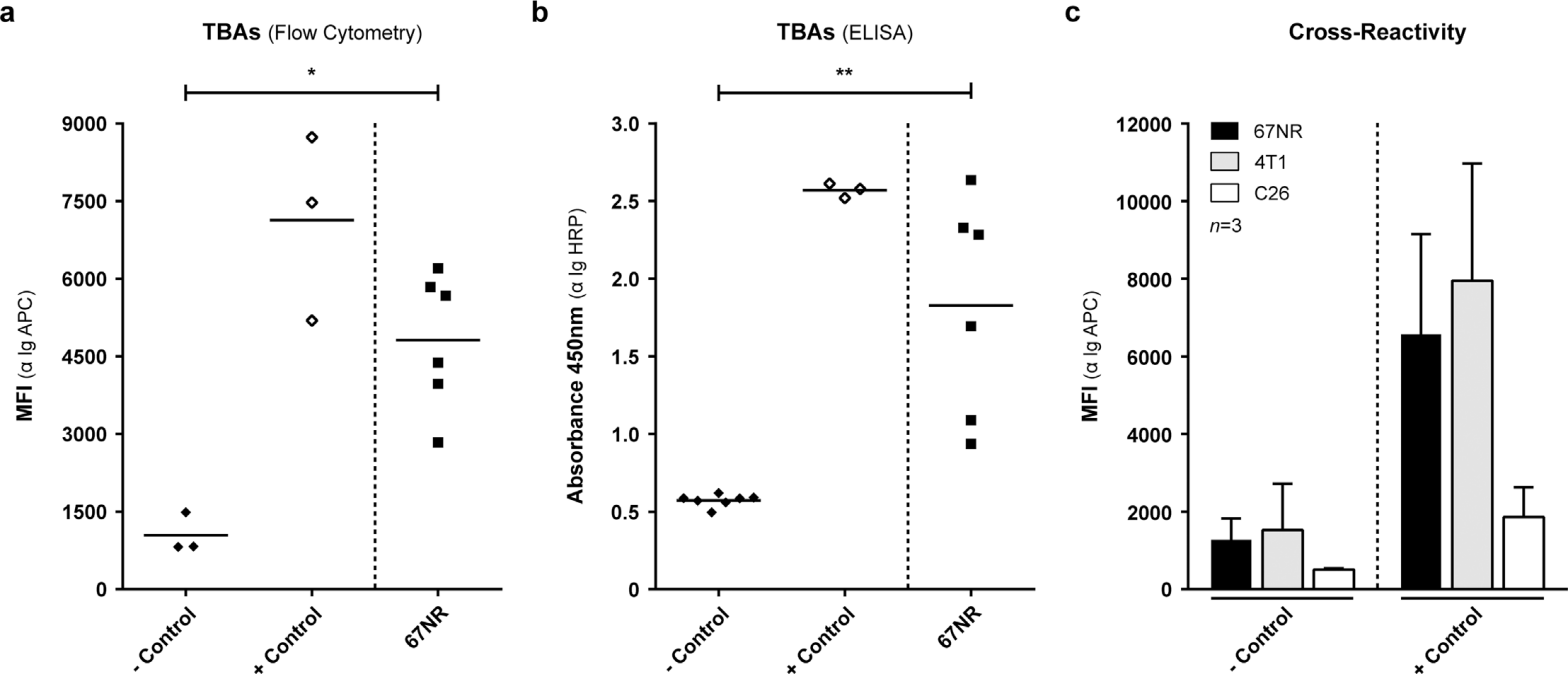
Detection and cross-reactivity of TBAs in the plasma of immunocompetent, vaccinated, and 67NR tumour-bearing Balb/C mice. a, b. Detection of TBAs in the plasma of immunocompetent (-control), vaccinated (+control), and 67NR tumour-bearing mice using cellular flow cytometry (α Ig APC) and ELISA (α Ig HRP) based systems. Mann-Whitney t-test with P<0.05 * and P<0.01 **. c. To determine whether the antibodies are 67NR specific, 1 *10^5^ murine 67NR, 4T1 (mammary), and C26 (colon) carcinoma cells were incubated with plasma from -control and +control Balb/C mice to determine the cross-reactivity of plasma containing antibodies. MFI: mean fluorescent intensity.

In order to investigate whether inoculation of the 67NR tumour itself results in the induction of antibodies we measured the immunoglobulin (Ig) levels in the plasma of tumour-bearing Balb/C mice using two detection methods. The optimization of both detection methods is shown in S2 and S3 Figs and these are further described in the supplementary materials and methods section (S1 File). Using these two independent methods we detected elevated levels of TBAs (MFI 4818 ±1306; 450nm 1.8 ±0.7) in tumour-bearing mice as compared to healthy immunocompetent (-control; MFI 1043 ±385; 450nm 0.6 ±0.04) mice (P<0.05 and P<0.01). As compared to the vaccinated mice (+control; MFI 7133 ±1795; 450nm 2.6 ±0.05), more variation was observed within the tumour-bearing mice (ELISA) but all animals showed increased TBAs as compared to healthy (-control) mice (Fig 3A and 3B).

In order to determine whether the detected antibodies were specific for 67NR, we incubated plasma from healthy immunocompetent (-control) and vaccinated (+control) mice with murine carcinoma cell lines 67NR, 4T1 (mammary), and C26 (colon) and visualized antibody binding by means of flow cytometric detection. The detected antibodies in the plasma of vaccinated Balb/C mice showed cross-reactivity with the 4T1 cell line (-control MFI 1530 ±1191; +control MFI 7947 ±3023), as the level of antibody binding was similar to the 67NR cell line (-control MFI 1278 ±546; +control MFI 6575 ±2575) (Fig 3C). In contrast, there was minimal antibody binding to the murine colon carcinoma C26 cell line indicating less cross reactivity (-control MFI 509 ±33; +control MFI 1862 ±764). The metastatic mammary carcinoma 4T1 cell line is a differentiated subclone of the non-metastatic 67NR cell line, which might clarify the similar binding of antibodies from ‘67NR’ vaccinated mice to 4T1 tumour cells.

Taken together these data indicate that 67NR inoculation is sufficient to drive the induction of a humoral immune response against the tumour in all mice. These antibodies bind specifically to the 67NR tumour and its related cell line 4T1 but not to the C26 cell line.

### Fractionated RT and DC stimulation did neither influence the levels nor the Ig isotype composition of TBAs in the plasma of tumour-bearing Balb/C mice

**Fig 4:**
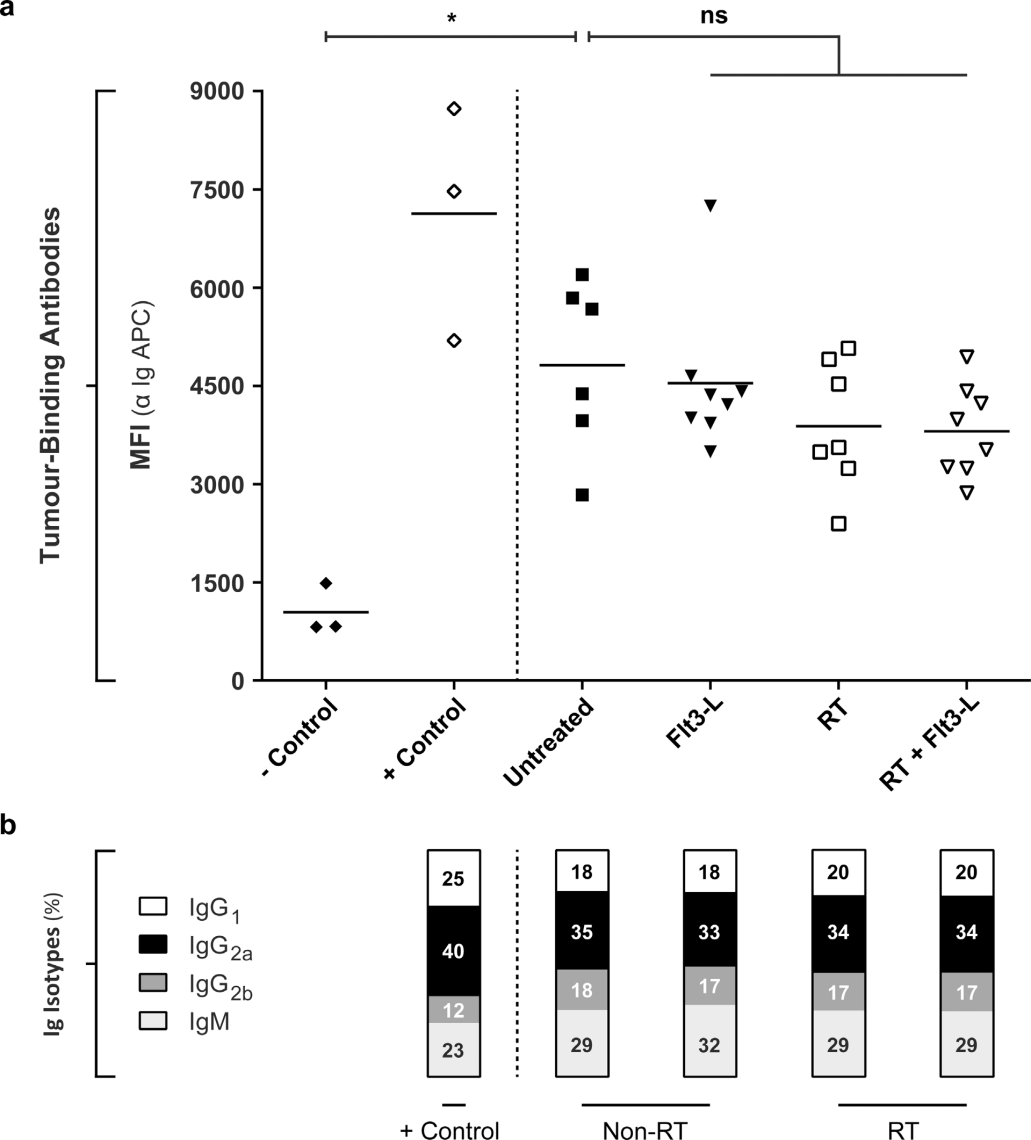
The levels and Ig isotype composition of TBAs in the plasma of (un)-treated mice. a. To assess the quantity of TBAs in the plasma, 1 *10^5^ 67NR tumour cells were incubated with plasma from immunocompetent (-control), vaccinated (+control), untreated, and treated (Flt3-L, RT, RT+Flt3-L) Balb/C mice. An APC conjugated antibody against total pan-Ig detected the TBAs. Mann-Whitney t-test with P<0.05 *. b. To determine the Ig isotype composition of the TBAs in the plasma, 1 *10^5^ 67NR tumour cells were incubated with plasma from vaccinated (+control), non-irradiated (non-RT: untreated, Flt3-L), and irradiated (RT: RT, RT+Flt3-L) Balb/C mice. The different isotypes were detected using fluorescent labelled IgG_1_, IgG_2a_, IgG_2b_, and IgM antibodies. The MFI values of the four Ig isotypes (S6 Fig) were combined and displayed as percentage of the total.

To assess whether the abscopal tumour growth inhibition that is observed after fractionated RT is paralleled with the modulation of the humoral anti-tumour response, we evaluated the quantity of TBAs in the plasma of sacrificed tumour-bearing mice that were either untreated or treated with Flt3-L, RT, or RT+Flt3-L. As shown in Fig 4A, we could not observe a difference in the total amount of TBAs between the experimental animal groups (Flt3-L MFI 4541 ±1148; RT MFI 3886 ±980; RT+Flt3-L MFI 3808 ±703) and the untreated (MFI 4818 ±1306) animal group indicating that the therapeutic effect of RT is not paralleled with an increase in the total amount of TBAs in the plasma. These findings were confirmed by ELISA (450nm: untreated 1.8 ±0.7; Flt3-L 1.6 ±0.3; RT 1.6 ±0.3; RT+Flt3-L 1.5 ±0.3) (S4A Fig).

Although we did not observe a quantitative difference between the experimental groups in the induction of antibodies, it remains possible that the Ig isotype composition of these TBAs is altered upon treatment. Because both detection assays utilize pan-Ig reactive secondary antibodies it is possible that the antibody response in the untreated group is an IgM dominated response whereas more polarized IgG responses are observed upon intervention. Using an optimized flow cytometric assay shown in S5 Fig and described in the supplementary materials and methods (S1 File), the T cell-dependent isotypes IgG_1_, IgG_2a_, IgG_2b_, and natural isotype IgM were measured in plasma of vaccinated (+control), untreated and treated (Flt3-L, RT, RT+Flt3-L) tumour-bearing mice. Vaccinated (+control) mice showed a different percentual composition of isotypes IgG (IgG_1_ 25%; IgG_2a_ 40%; IgG_2b_ 12%) and IgM (23%) as compared to tumour-bearing mice in non-RT and RT groups (IgG_1_ 19 ±1.2%; IgG_2a_ 34 ±0.8%; IgG_2b_ 17 ±0.5%; IgM 30 ±1.5%) (Fig 4B). In particular, IgG_1_ and IgG_2a_ were induced upon vaccination whereas IgG_2b_ and IgM were decreased in relative percentage as compared to the other isotypes. However, they were not reduced in absolute MFI (S6 Fig). Notably we did neither observe differences in percentual composition of the Ig isotypes, nor in the quantity (IgG_1_ MFI 3976 ±661; IgG_2a_ MFI 7039 ±1011; IgG_2b_ MFI 3533 ±479; IgM MFI 6180 ±1940) (S6 Fig) between the tumour-bearing mice in the different treatment groups.

### Immunoglobulins, B cells, and T cells were present in the primary and secondary tumour

**Fig 5:**
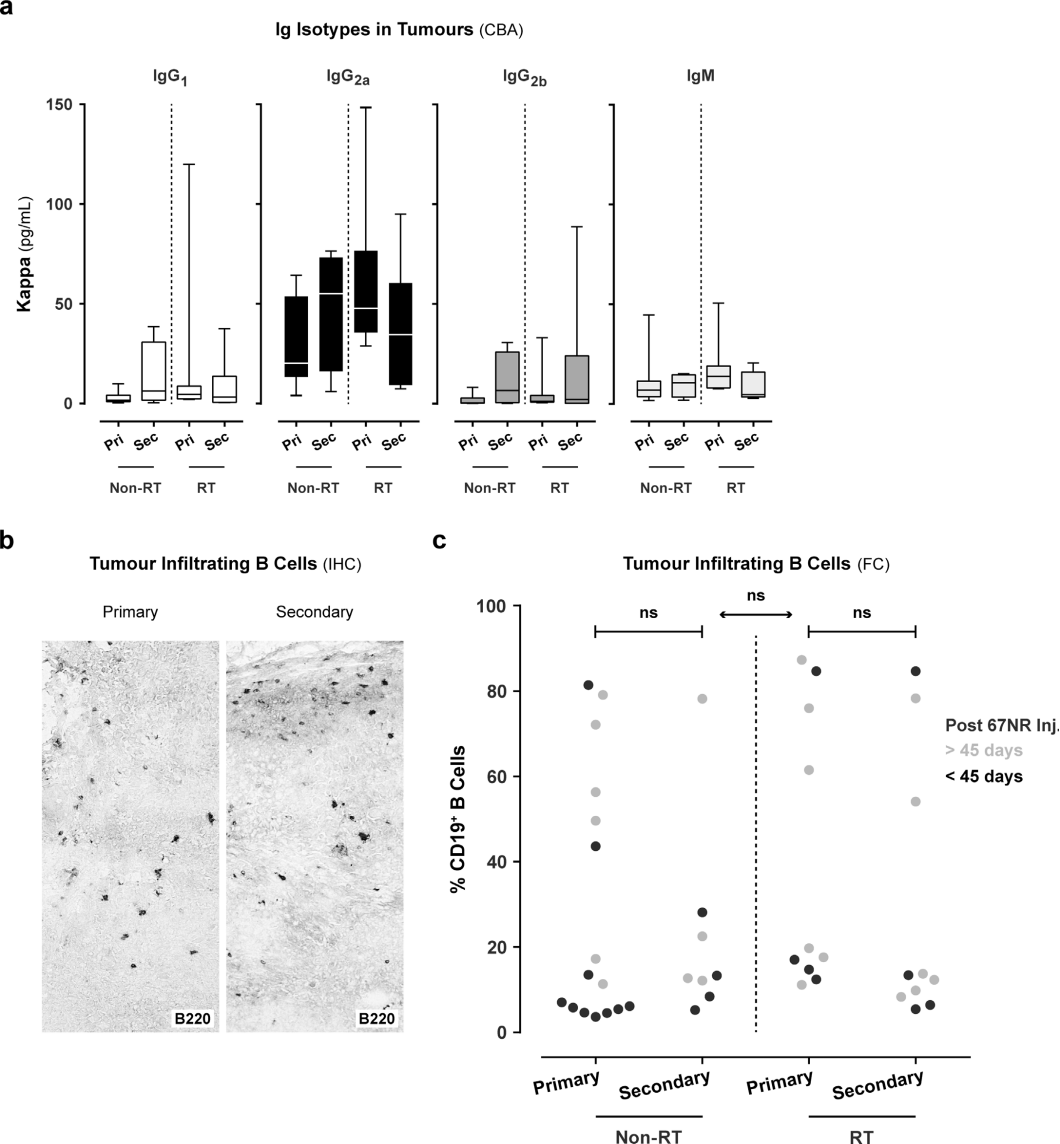
Infiltration of immunoglobulins and B cells in the primary and secondary tumour. a. To determine the quantity of immunoglobulin (Ig) isotypes (IgG_1_, IgG_2a_, IgG_2b_, and IgM) in both tumours, we used a cytometric bead assay (CBA). The isotype quantity was determined using a standard curve per isotype. These values were corrected for total protein content of the tumour lysates and displayed as μg/mL for isotypes kappa IgG_1_, IgG_2a_, IgG_2b_, and IgM. b. Immunohistochemical (IHC) staining of tumour infiltrating B cells (B220) in the primary and secondary tumour (data representative for all animals). c. Detection of B cells in the primary and secondary tumours using flow cytometry (FC). Gating strategy: Living haematopoietic cells (CD45+ vs. 7-AAD-) were gated. In this gate, the lymphocyte population was selected based on the forward- (FSC) vs. sideward (SSC) scatter. To ensure that only lymphocytes were gated, CD11c+ cells were excluded. The CD19+ B cells were selected in the CD11c- gate. The percentage CD19+ B cells in the primary and secondary tumour of (non)- vs. irradiated animals (black or grey, respectively < 45 or > 45 days after 67NR tumour inoculation). Mann-Whitney t-test.

In order to determine whether antibodies and immune cells are present at the site of the tumour, we measured the presence and Ig isotype composition (T cell-dependent isotypes IgG_1_, IgG_2a_, IgG_2b_, and natural isotype IgM) of TBAs in homogenized tumour tissues using a cytometric bead assay. We detected Ig isotypes IgG_1_, IgG_2a_, IgG_2b_, and IgM in the primary and secondary tumour of mice in non-RT and RT groups (Fig 5A). Both irradiated and non-irradiated tumour-bearing mice showed an increased quantity of kappa IgG_2a_ (65 ±46 pg/mL) as compared to kappa IgG_1_ (14 ±26 pg/mL), IgG_2b_ (12 ±22 pg/mL) and IgM (17 ±14 pg/mL). The Ig isotypes kappa IgG_3_, IgA, and IgE were absent in both tumours (data not shown).

In addition, we also evaluated the presence of B cells (and T cells) in the tumours. First, we evaluated the localisation of these cells using IHC. Therefore, we performed IHC and visualized B cells and CD3+, CD4+, and CD8+ T cells in both the primary and secondary tumours (Fig 5B; S7A Fig). In none of the evaluated IHC tumour specimens we observed the presence of B or T cells in a tertiary lymphoid structure. We only observed solitary B cells and T cells that infiltrated throughout the tumour tissue or small clusters around the tumour tissue.

Second, in order to quantitate the infiltration of CD19+ B cells and CD4+, CD8+ T cells we performed flow cytometric stainings on single tumour cell suspensions. Using this approach, we confirmed and quantitated the presence of CD19+ B cells as well as CD4+, CD8+ T cells in the tumour at the end of the experiment (Fig 5C; S7B–S7D Fig). Notably, in a subset of tumour specimens we observed that B cells predominated the immune infiltrate accounting up to 90% of the infiltrate. This was not correlated with higher TBA titers in the plasma. Furthermore, our flow cytometric analysis of the infiltrate at the human endpoint of the experiment showed that T cells are more abundant in the infiltrate in non-irradiated tumours (CD4+ 14 ±11%; CD8+ 19 ±14%) as compared to irradiated tumours (CD4+ 5 ±2%; CD8+ 8 ±11%) (S7C and S7D Fig).

Taken together these data indicate that we did not find evidence indicating tertiary lymphoid structures or local production of TBAs at site of the tumours. In cases where primary tumours could be collected in the irradiated animals at the end of the experiment there were less T cells infiltrating the tumour as compared to the primary tumours of non-irradiated animals. It should, however, be emphasized that this evaluation takes place after reaching the endpoint of the experiment that is often characterized by exponential growth of the tumour. In cases where one of the tumours was resolved these specimens could not be collected.

## Discussion

Undebatable T cells play a crucial role in abscopal tumour regression as illustrated by several lines of evidence in literature. The most compelling proof of concept was the demonstration of Demaria *et al*. that abscopal tumour regression depended totally on the presence of T cells [23]. In subsequent research, Dewan *et al*. further showed that predominantly cytotoxic CD8+ T cells were associated with abscopal tumour regression [24]. However, in human individuals demonstrating abscopal tumour regression data were gathered implicating the involvement of additional immune mechanisms [10, 27]. Certain immunological phenomena like humoral immune responses may be involved in tumour control or serve as a biomarker identifying responding patients.

Based on two clinical case studies that reported an association between abscopal tumour regression and the induction of humoral responses [10, 11], we set out to analyse the humoral immune response in an animal model (67NR mammary carcinoma) showing abscopal effects [23]. We observed that whereas a booster vaccine was necessary to induce durable antibody responses against heat-killed tumour cells in Balb/C mice, all mice that were inoculated with a 67NR tumour showed an antibody response against the tumour. This observation together with the previously reported pre-existing antibody response in patients displaying abscopal tumour regression triggers the question whether such a pre-existing antibody response is a prerequisite for responses against metastatic disease [27]. Regardless of the direct role of such antibodies in the control of disease their mere presence indicates that the tumour has not escaped immune recognition.

Based on our findings we cannot specify the antigens that are expressed on the cell surface of 67NR tumours. Presumably the antibodies in the plasma bind to epitopes that are shared between various tumours that target tumour-associated rather than tumour-specific antigens. Nevertheless these antigens are clearly more abundant on 67NR tumour cells and its derived cell line 4T1 selected for being more metastatic [29, 30] as compared to the colon carcinoma C26 cell line, which could reflect a certain contribution of antibodies targeting tumour-specific antigens. Furthermore, we observed that these durable antibody responses were not limited to an IgM profile, but were quite diverse in terms of the Ig isotypes, which is most likely a reflection that multiple antigens are recognized during 67NR tumour progression. Additionally, the fact that the antibody response is dominated by IgG can be considered as direct proof that CD4+ T cell-dependent responses are triggered upon tumour inoculation as immunoglobulin class switching occurred [31]. Currently, it remains unknown whether the induction of antibody responses against the tumour is directly involved in abscopal tumour regression, or whether the induction of such antibodies only serves as a biomarker indicating a novel immune response in a subset of patients.

In order to address a potential role of this humoral immune response we evaluated how this was altered upon fractionated RT. We showed that abscopal tumour growth inhibition is observed after fractionated RT in the 67NR mouse model. Fractionated RT in combination with or without Flt3-L administration resulted in growth delay of both irradiated and non-irradiated tumours, indicating abscopal effects. The groups receiving RT also showed an increased number of surviving tumour free mice. However, several of these irradiated tumour-bearing mice demonstrated exponential tumour growth, suggesting the importance of the immunogenic state of the Balb/C mice as well as the 67NR tumour. We applied fractionated RT to the primary tumour to reduce harmful side effects to the surrounding healthy tissue for clinical translation. This approach resulted in abscopal effects (RT groups), which was also observed by Dewan *et al*. They demonstrated that fractionated RT was more effective than a single dose of RT. This latter finding is in line with previous research of Demaria *et al*. who indicated that a single dose of RT did not result in abscopal effects [23, 24]. Importantly, it should be noted that all depends on the trigger dose of RT that is applied, if this is large enough abscopal effects could be seen. In addition, our objective of the Flt3-L group was to evaluate whether stimulating pDC and cDC might be effective at boosting immune responses that can be read out via monitoring the B cells. Notably, the tumour growth inhibition we observed in irradiated groups in presence or absence of Flt3-L was not paralleled with the modulation of the pre-existing antibody response. Likewise, we demonstrated that DC stimulation with Flt3-L did neither modulate the magnitude nor the composition of the humoral immune response. In contrast, preclinical studies showed an increased anti-tumour effect mediated by T cells using the same Flt3-L concentrations and treatment days [23, 32]. Notably, we showed that administration of Flt3-L had no additive effect in humoral immune responses. Finally, we found no differences in immune infiltrate and Ig isotype content of all tumours that were indicative for local Ig production. Because the main endpoint of our study was observing the survival benefit we did not sacrifice animals at the time of abscopal tumour shrinkage, but only at the predefined human endpoint or at the end of the experiment. Furthermore, the aim of the histology and flow cytometry of the tumour was to exclude that there was local antibody production at the site of the tumour. This study design does presumably not allow the identification of the mechanism leading to abscopal tumour shrinkage. For this reason, we observed the differences in T cell infiltration of non-irradiated and irradiated tumours.

The lack of modulating the antibody response upon RT suggests that antibodies do not play a key role in abscopal tumour regression in the 67NR mouse model. However, current immunotherapy approaches that use monoclonal antibodies targeting immune checkpoints show significant anti-tumour responses in multiple cancer types, so monitoring B cell- as well as antibody responses might be of great value [33]. Although, Ig isotype classification in the 67NR mouse model indicated that the TBAs were predominantly IgG_2a_, which is the most effective at complement fixation and antibody-dependent cellular cytotoxicity (ADCC) [34–36], it is also well documented that tumour cells have several evading mechanisms for antibody-mediated complement-dependent cytotoxicity (CDC) by the expression of membrane complement regulatory proteins (mCRPs) and proteases degrading complement at the cell surface that may interfere with the efficacy of the antibody-mediated killing [37]. This is in line with Demaria *et al*. who showed the crucial role of T cells in abscopal tumour regression [23, 24]. Furthermore, the lack of correlation between the occurrence of abscopal tumour regression and modulation of the humoral immune response in our 67NR mouse model also indicates that monitoring the humoral immune response is not a surrogate marker that is invariably associated with the induction of systemic immune responses. Golden *et al*. demonstrated in a subset of patients with abscopal effects that not only the pre-existing antibody response was enhanced in magnitude but also antibodies targeting different epitopes of the same antigen and seromic analysis demonstrated that in one patient a novel antibody response against ten new antigens was observed [10]. Based on the contradicting results demonstrated in our study using an animal model inoculated with a single tumour cell line and the clinical case reports it remains too early to assess to what extent monitoring the course of the antibody response can be applicable in the clinic for monitoring abscopal immune responses. So, in future more preclinical work should be performed to evaluate the antibody response in multiple types of cancer that demonstrate abscopal effects and if these TBAs correlate with responders that show (abscopal) tumour regression.

Until now abscopal tumour regression after RT alone is only anecdotally observed in patients with cancer. However, recent studies have demonstrated that combination therapy with fractionated RT and CTLA-4 blockade (ipilimumab) or GM-CSF treatment, the percentage of patients showing abscopal tumour regression increased up to 20% [10, 13, 27]. Importantly, accumulating evidence demonstrates that in particular RT in combination with immune modulation induces local and systemic anti-tumour responses and future approaches should be evaluated to boost the link between the innate and adaptive immune systems to increase the occurrence of abscopal effects [38, 39]. To this end, the stimulation of pDC and cDC by immunotherapeutic agents like toll-like receptor (TLR) agonists could enhance effector T cell priming [40]. In addition, immune checkpoint inhibitors like CTLA-4, the PD-1/PD-L1 axis, and CD40 could overcome the suppressive microenvironment of the tumour, and prevent T cell exhaustion and apoptosis [33, 41–43]. The blockade of these particular immune checkpoints with monoclonal antibodies promotes the endogenous anti-tumour activation of immune cells that resulted in significant benefits for cancer patients [44]. With respect to CTLA-4, Dewan *et al*. showed CD8+ T cell-mediated abscopal effects in a pre-clinical animal model in which fractionated RT was combined with CTLA-4 blockade [24]. It might be of value to study the relevance of antibodies in these treatment systems.

In conclusion, our study showed that fractionated RT with or without Flt3-L administration induces abscopal effects in the 67NR mouse model. Here, we observed a pre-existing antibody response that is also observed in abscopal responders in human studies. However, such humoral immune responses in terms of total quantity of plasma antibodies or the Ig isotype composition are not altered upon fractionated RT and/or upon DC stimulation with Flt3-L in this model. Taken together our data demonstrate that monitoring antibodies in the setting of abscopal tumour regression does not invariably associates with therapeutic effects.

## Supporting Information

S1 Fig The tumour volume in mm^3^ of the primary and secondary tumour in Balb/C mice 14 days after 67NR inoculation. The size (mm^3^) of both 67NR tumours in Balb/C mice assigned to groups untreated, Flt3-L, RT, and RT+Flt3-L. All Balb/C mice had palpable tumours at the start of the experiment (before the treatments). The stars indicate Balb/C mice that were tumour free at the end of the experiment (day 162).
(TIF)

S2 Fig Experimental optimization of a flow cytometric detection system to analyse immunoglobulins in the plasma of Balb/C mice. a. The surface expression of H-2K^d^ (MHC-I) on 67NR tumour cells. An APC-conjugated antibody against total immunoglobulins (α Ig APC) detected the un-conjugated H-2K^d^ antibody (0.5 mg/mL) using flow cytometry (FC). In order to quantitatively analyse the antibody concentration without the influence of matrix effects that originate from undesired protein factors in the plasma, we performed a dilution experiment of an antibody of known concentration in different concentrations of plasma. We stained 10^5^ cells with 5 concentrations (0.5, 0.1, 0.05, 0.01, 0.005 μg/μL) of a monoclonal unconjugated H-2K^d^ antibody in 5 concentrations of mouse plasma (1:2, 1:10, 1:20, 1:40, 1:80) or in buffer. b. Different numbers (0.75 *10^5^ to 10 *10^5^) of 67NR tumour cells were incubated with vaccinated (+control) or -control plasma and detected using an α Ig APC antibody to determine the optimal signal to background ratio. Iso: Ig isotype control, MFI: mean fluorescent intensity.
(TIF)

S3 Fig Vaccine-induced antibody response in immunocompetent Balb/C mice. Balb/C (immunocompetent) mice were vaccinated with incomplete Freund’s adjuvant (IFA), CpG oligodeoxynucleotides (ODNs), and heat-killed 67NR cells. To boost the antibody response, a secondary vaccination cocktail of IFA, CpG ODNs, and heat-killed 67NR cells was injected in primary immunized Balb/C mice. Per week one injected mouse was sacrificed and the plasma was collected (technical replicates). The levels of immunoglobulins (α Ig APC) in the plasma were measured after one injection (week 3, 4, 5) or boost injection (week 1, 2, 3). The plasma samples from the boosted mice were used as positive control in the cellular flow cytometry and ELISA-based detection systems. Control immunocompetent Balb/C mice were injected with phosphate buffered saline (PBS).
(TIF)

S4 Fig Detection of TBAs in the plasma of (un)-treated Balb/C mice using an optimized cellular ELISA based system. a. To determine the optimal concentration of the detection antibody, we titrated the α Ig HRP antibody (1:2000, 1:4000, 1:8000) using vaccinated and -control plasma. To control for background staining we included the α Ig HRP only and unstained. b. After determining the optimal α Ig HRP dilution, the plasma of vaccinated and -control was titrated (1:20, 1:40, 1:80, 1:160, 1:320) and detected using a 1:2000 dilution of α Ig HRP. The background staining was determined as described previously. c. A total of 1 *10^5^ 67NR tumour cells were coated and incubated with plasma from immunocompetent (-control), vaccinated (+control), untreated, and treated (Flt3-L, RT, RT+Flt3-L) Balb/C mice. An HRP conjugated antibody against total Ig detected the TBAs. Tumour-bearing mice received fractionated RT (RT, RT+Flt3-L) or no RT (untreated, Flt3-L). Mann-Whitney t-test with P<0.01 **.
(TIF)

S5 Fig No cross-reactivity of specific mouse anti-Ig isotype antibodies. a. Schematic representation of the optimization procedure. Freshly isolated human peripheral blood mononuclear cells (PBMCs) were incubated with unconjugated mouse anti-human antibodies specific for CD28 (IgG_1_), CD3 (IgG_2a_), CD158b (IgG_2b_), and CD158a (IgM). These mouse anti-human antibodies were detected with an antibody cocktail containing rat anti-mouse IgG_1_ (APC), IgG_2a_ (PE), IgG_2b_ (FITC), and IgM (PE-Cy7). b. FC dotplots of the gating strategy. The lymphocyte population was gated in the FSC vs. SSC dotplot. The positive IgG_1_ population was negative for isotypes IgG_2a_, IgG_2b_, and IgM, indicating no cross-reactivity. This strategy was repeated for all Ig isotypes. c. The MFI levels of isotypes IgG_1_, IgG_2a_, IgG_2b_, and IgM. These rat anti-mouse Ig isotype antibodies show minimal crossreactivity.
(TIF)

S6 Fig The Ig isotype composition of the TBAs in the plasma of tumour-bearing Balb/C mice displayed as MFI values. To determine the Ig isotypes in the plasma of tumour-bearing mice, 1 *10^5^ 67NR tumour cells were incubated with plasma from vaccinated (+control), non-irradiated (untreated, Flt3-L), and irradiated (RT, RT+Flt3-L) Balb/C mice. The -control plasma was used to assess the background of each isotype staining. The four different isotypes were detected using fluorescent labelled IgG_1_ (APC), IgG_2a_ (PE), IgG_2b_ (FITC), and IgM (PE-Cy7) antibodies.
(TIF)

S7 Fig Infiltration of CD4+ and CD8+ T cells in the primary and secondary tumour. a. Immunohistochemical (IHC) staining of CD3+, CD4+, and CD8+ tumour infiltrating T cells in both the primary and secondary tumour (data representative for all animals). b. FC density plots of the gating strategy. Living haematopoietic cells (CD45+ vs. 7-AAD-) were gated. In this gate, the lymphocyte population was selected based on the FSC vs. SSC. To ensure that only lymphocytes were gated, CD11c+ cells were excluded. In the CD11c- (CD8α) gate, CD4+ and CD8α+ T cells were selected. c, d. The percentage CD4+ and CD8α+ T cells in the primary and secondary tumour of (non)- vs. irradiated animals measured with FC.
(TIF)

S1 File Supplementary materials and methods. Development of cellular read-out systems for the detection of TBAs in the plasma of Balb/C mice.
(PDF)
