# Supplementary figures and images for: Fractionated Radiotherapy with 3 x 8 Gy Induces Systemic Anti-Tumour Responses and Abscopal Tumour Inhibition without Modulating the Humoral Anti-Tumour Response
Source: PLoS One. 2016 Jul 18;11(7):e0159515. doi: 10.1371/journal.pone.0159515 (PMC4948777; doi:10.1371/journal.pone.0159515)

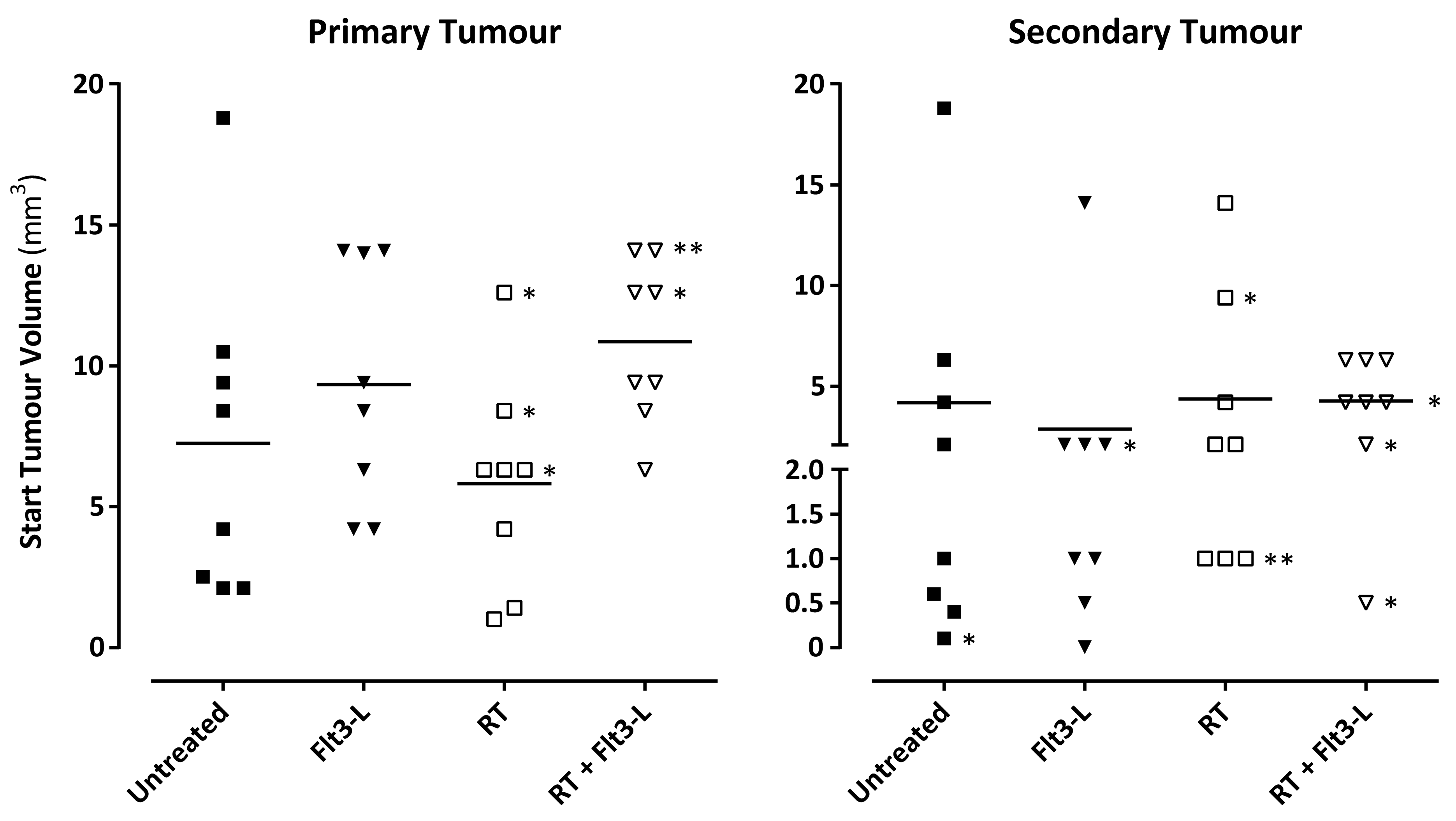

Supplement: S1 Fig — The size (mm3) of both 67NR tumours in Balb/C mice assigned to groups untreated, Flt3-L, RT, and RT+Flt3-L. All Balb/C mice had palpable tumours at the start of the experiment (before the treatments). The stars indicate Balb/C mice that were tumour free at the end of the experiment (day 162). (TIF) [file pone.0159515.s001.tif]

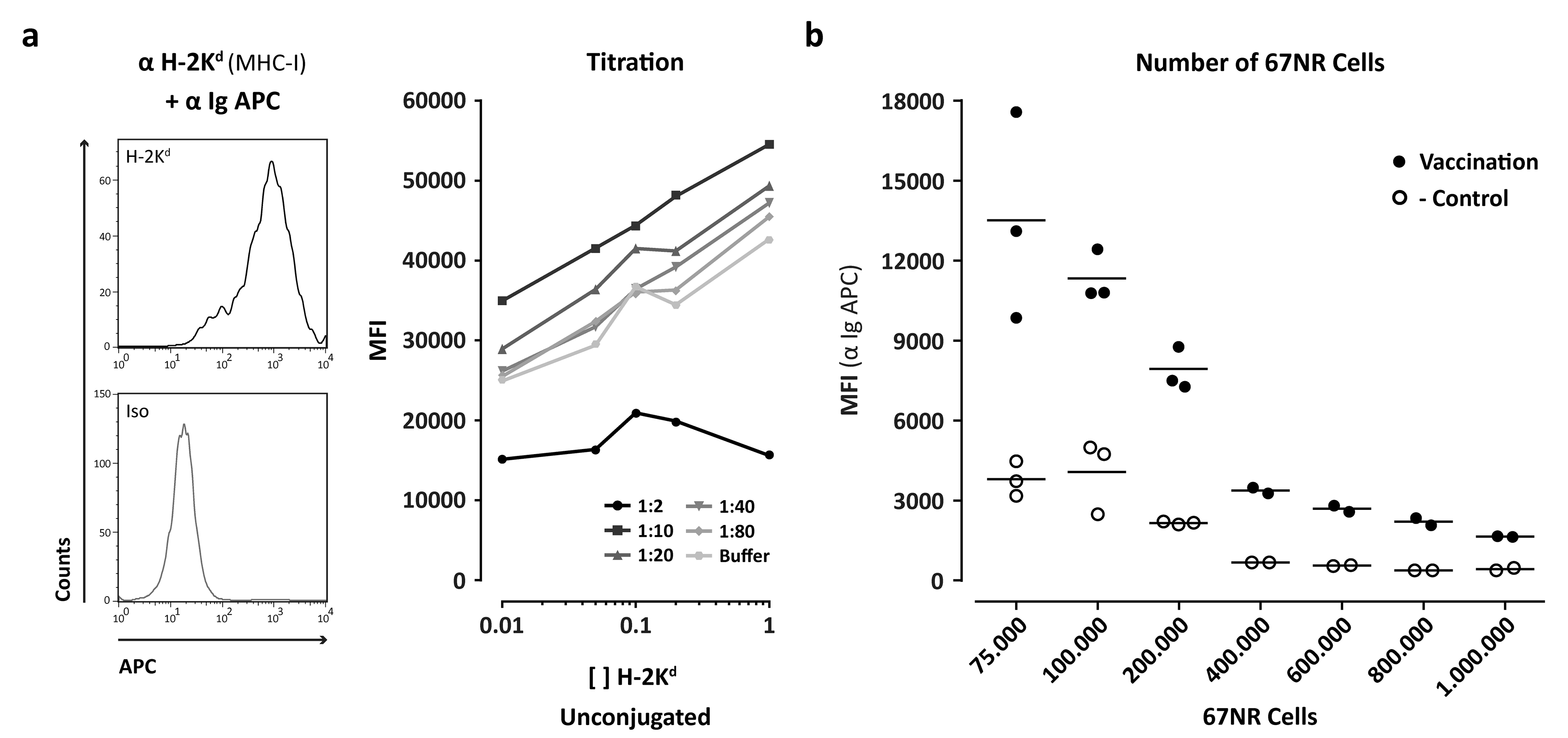

Supplement: S2 Fig — a. The surface expression of H-2Kd (MHC-I) on 67NR tumour cells. An APC-conjugated antibody against total immunoglobulins (α Ig APC) detected the un-conjugated H-2Kd antibody (0.5 mg/mL) using flow cytometry (FC). In order to quantitatively analyse the antibody concentration without the influence of matrix effects that originate from undesired protein factors in the plasma, we performed a dilution experiment of an antibody of known concentration in different concentrations of plasma. We stained 105 cells with 5 concentrations (0.5, 0.1, 0.05, 0.01, 0.005 μg/μL) of a monoclonal unconjugated H-2Kd antibody in 5 concentrations of mouse plasma (1:2, 1:10, 1:20, 1:40, 1:80) or in buffer. b. Different numbers (0.75 *105 to 10 *105) of 67NR tumour cells were incubated with vaccinated (+control) or -control plasma and detected using an α Ig APC antibody to determine the optimal signal to background ratio. Iso: Ig isotype control, MFI: mean fluorescent intensity. (TIF) [file pone.0159515.s002.tif]

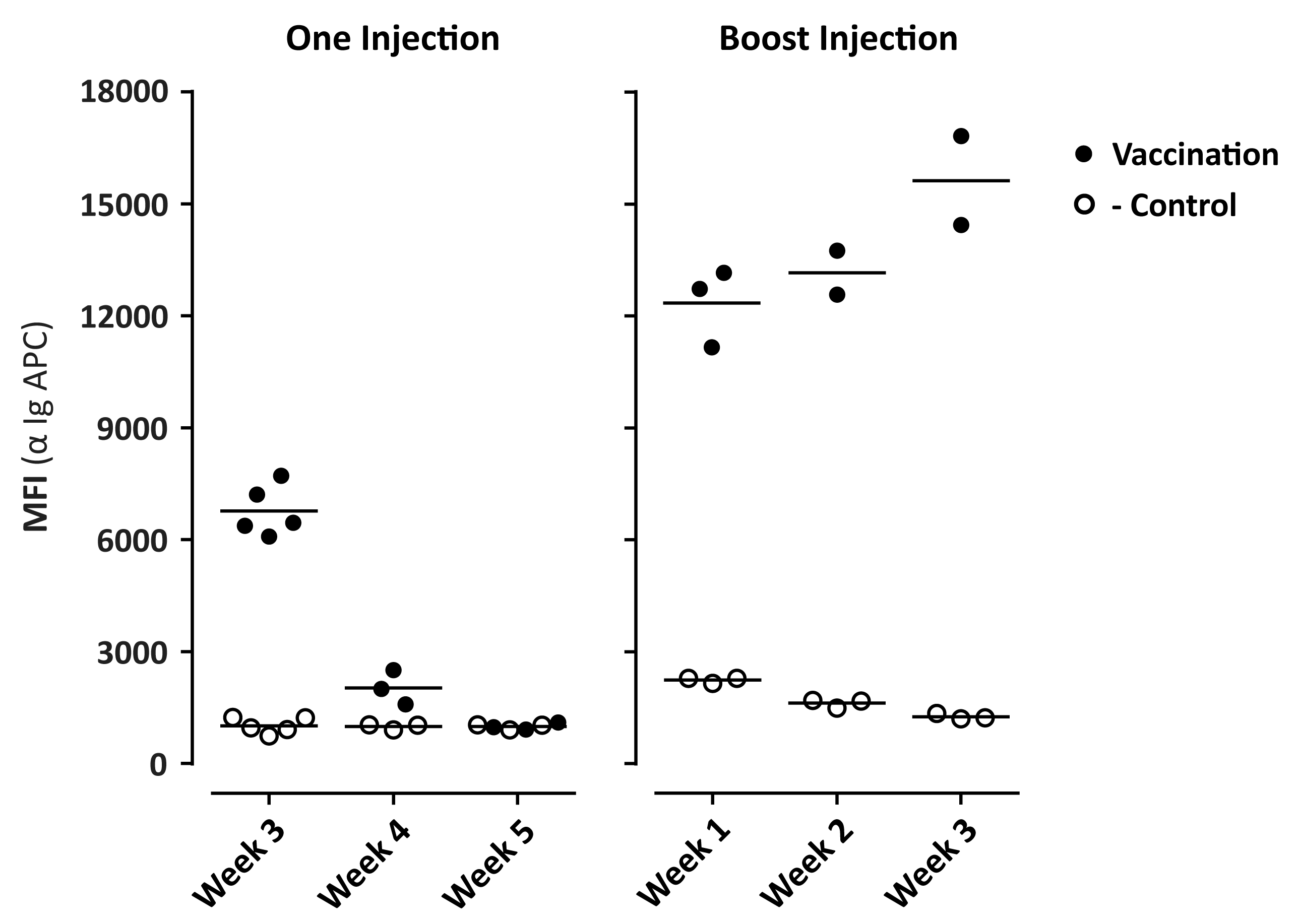

Supplement: S3 Fig — Balb/C (immunocompetent) mice were vaccinated with incomplete Freund’s adjuvant (IFA), CpG oligodeoxynucleotides (ODNs), and heat-killed 67NR cells. To boost the antibody response, a secondary vaccination cocktail of IFA, CpG ODNs, and heat-killed 67NR cells was injected in primary immunized Balb/C mice. Per week one injected mouse was sacrificed and the plasma was collected (technical replicates). The levels of immunoglobulins (α Ig APC) in the plasma were measured after one injection (week 3, 4, 5) or boost injection (week 1, 2, 3). The plasma samples from the boosted mice were used as positive control in the cellular flow cytometry and ELISA-based detection systems. Control immunocompetent Balb/C mice were injected with phosphate buffered saline (PBS). (TIF) [file pone.0159515.s003.tif]

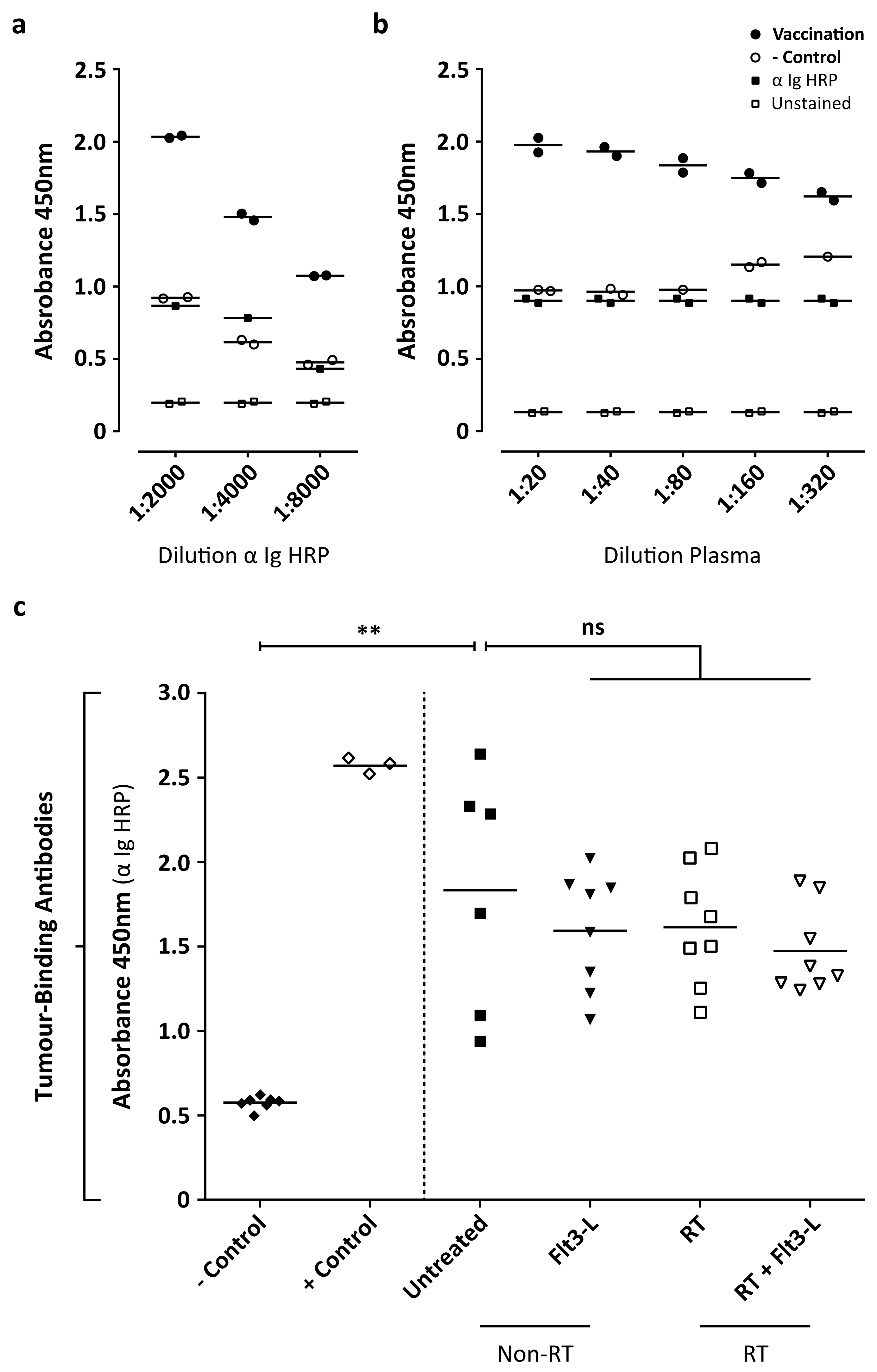

Supplement: S4 Fig — a. To determine the optimal concentration of the detection antibody, we titrated the α Ig HRP antibody (1:2000, 1:4000, 1:8000) using vaccinated and -control plasma. To control for background staining we included the α Ig HRP only and unstained. b. After determining the optimal α Ig HRP dilution, the plasma of vaccinated and -control was titrated (1:20, 1:40, 1:80, 1:160, 1:320) and detected using a 1:2000 dilution of α Ig HRP. The background staining was determined as described previously. c. A total of 1 *105 67NR tumour cells were coated and incubated with plasma from immunocompetent (-control), vaccinated (+control), untreated, and treated (Flt3-L, RT, RT+Flt3-L) Balb/C mice. An HRP conjugated antibody against total Ig detected the TBAs. Tumour-bearing mice received fractionated RT (RT, RT+Flt3-L) or no RT (untreated, Flt3-L). Mann-Whitney t-test with P<0.01 **. (TIF) [file pone.0159515.s004.tif]

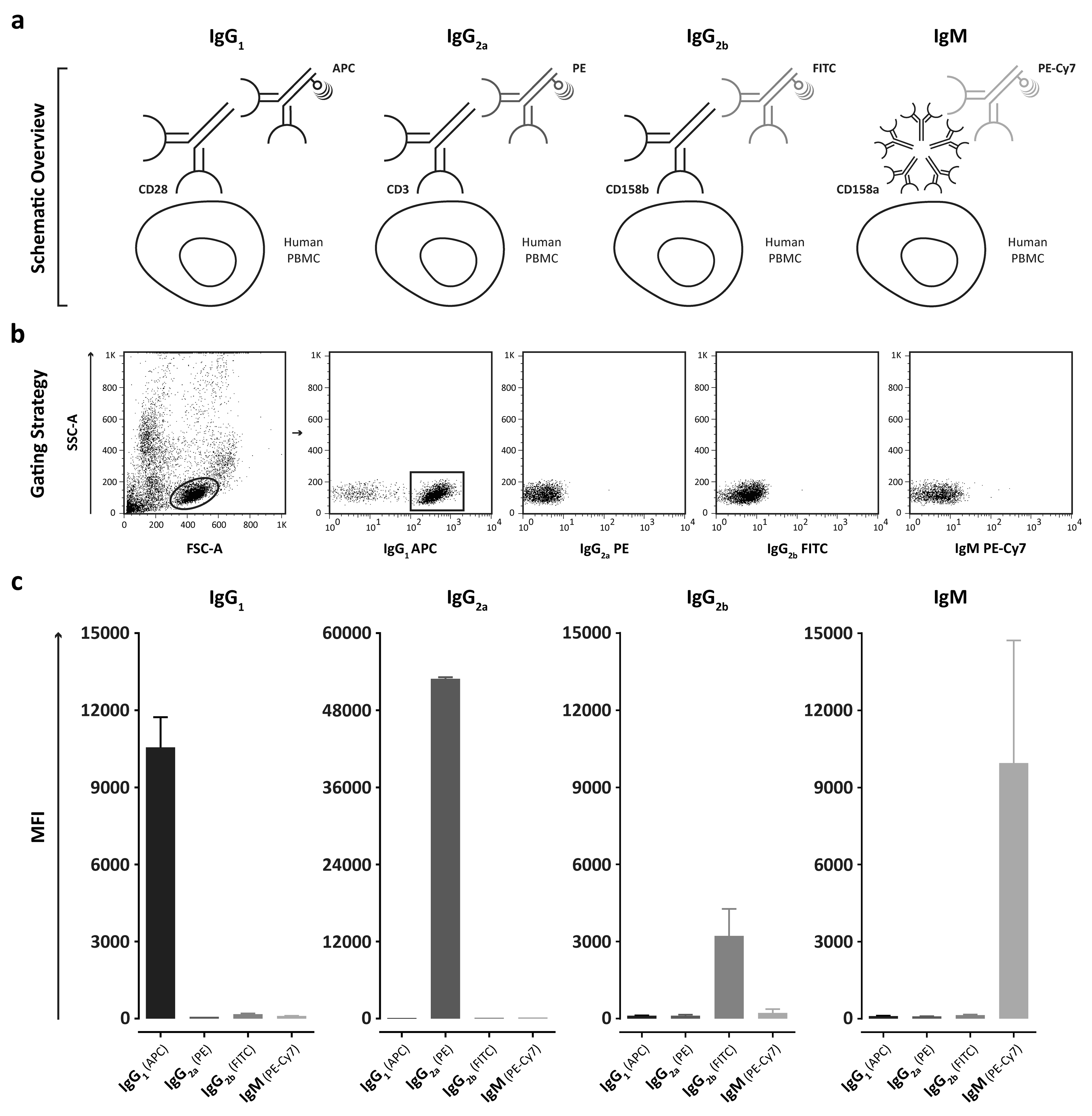

Supplement: S5 Fig — a. Schematic representation of the optimization procedure. Freshly isolated human peripheral blood mononuclear cells (PBMCs) were incubated with unconjugated mouse anti-human antibodies specific for CD28 (IgG1), CD3 (IgG2a), CD158b (IgG2b), and CD158a (IgM). These mouse anti-human antibodies were detected with an antibody cocktail containing rat anti-mouse IgG1 (APC), IgG2a (PE), IgG2b (FITC), and IgM (PE-Cy7). b. FC dotplots of the gating strategy. The lymphocyte population was gated in the FSC vs. SSC dotplot. The positive IgG1 population was negative for isotypes IgG2a, IgG2b, and IgM, indicating no cross-reactivity. This strategy was repeated for all Ig isotypes. c. The MFI levels of isotypes IgG1, IgG2a, IgG2b, and IgM. These rat anti-mouse Ig isotype antibodies show minimal crossreactivity. (TIF) [file pone.0159515.s005.tif]

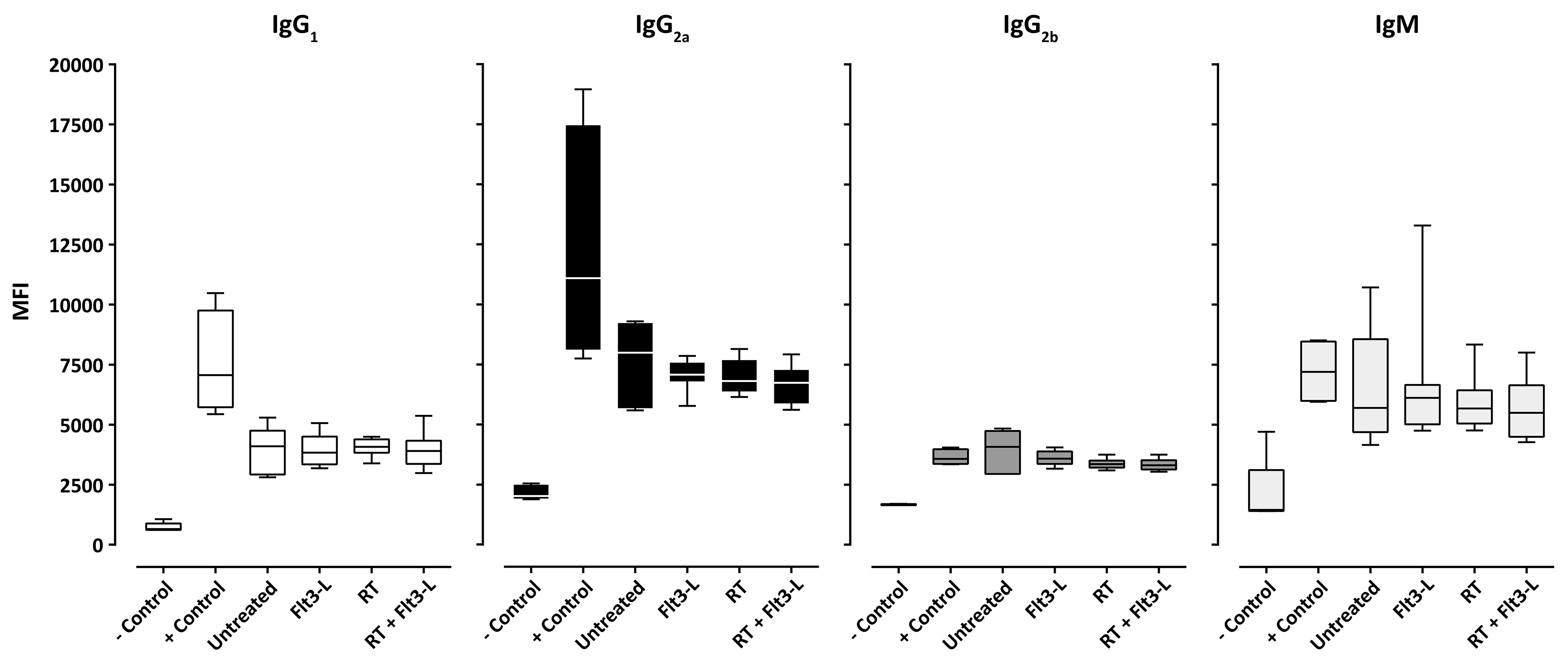

Supplement: S6 Fig — To determine the Ig isotypes in the plasma of tumour-bearing mice, 1 *105 67NR tumour cells were incubated with plasma from vaccinated (+control), non-irradiated (untreated, Flt3-L), and irradiated (RT, RT+Flt3-L) Balb/C mice. The -control plasma was used to assess the background of each isotype staining. The four different isotypes were detected using fluorescent labelled IgG1 (APC), IgG2a (PE), IgG2b (FITC), and IgM (PE-Cy7) antibodies. (TIF) [file pone.0159515.s006.tif]

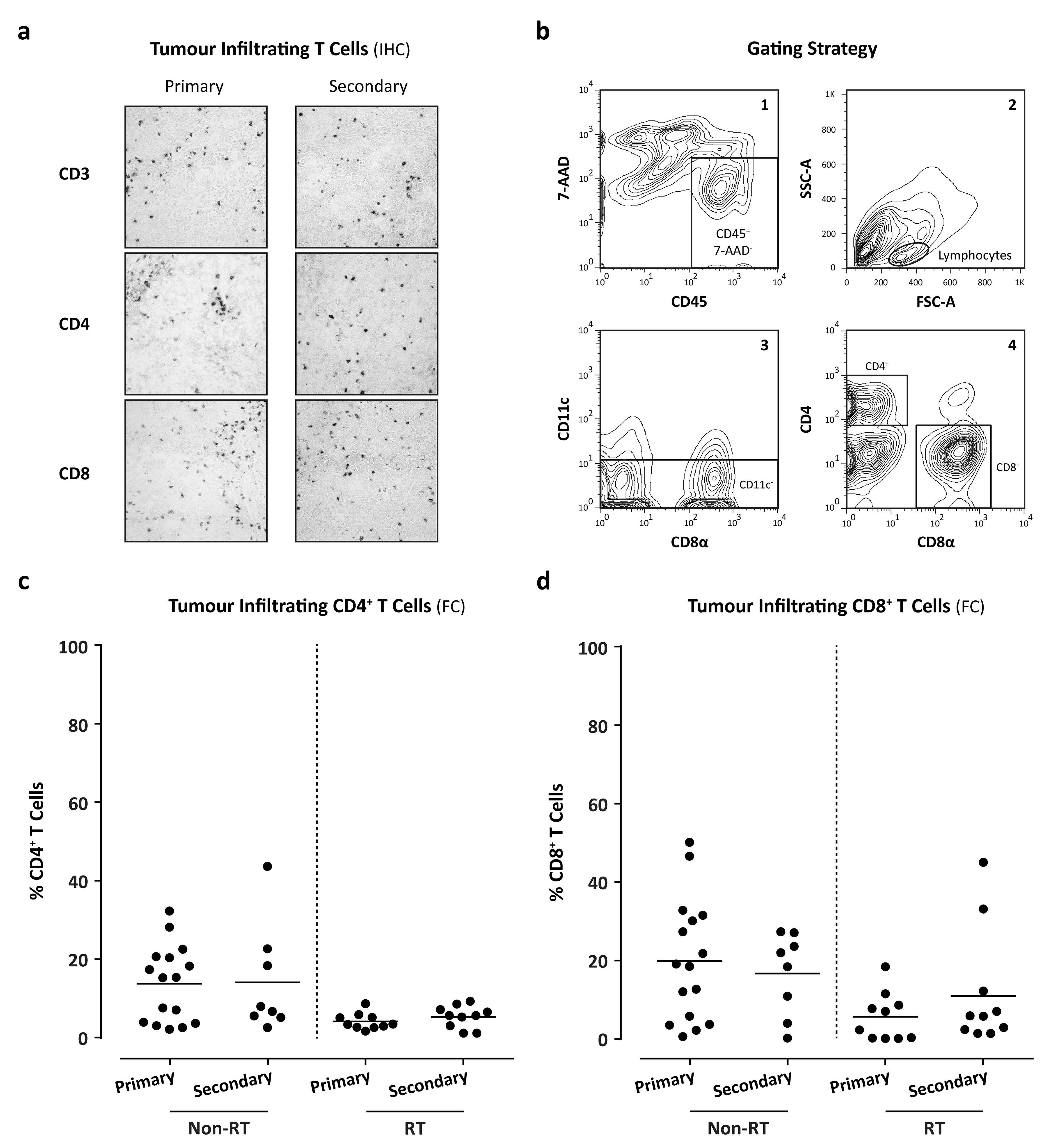

Supplement: S7 Fig — a. Immunohistochemical (IHC) staining of CD3+, CD4+, and CD8+ tumour infiltrating T cells in both the primary and secondary tumour (data representative for all animals). b. FC density plots of the gating strategy. Living haematopoietic cells (CD45+ vs. 7-AAD-) were gated. In this gate, the lymphocyte population was selected based on the FSC vs. SSC. To ensure that only lymphocytes were gated, CD11c+ cells were excluded. In the CD11c- (CD8α) gate, CD4+ and CD8α+ T cells were selected. c, d. The percentage CD4+ and CD8α+ T cells in the primary and secondary tumour of (non)- vs. irradiated animals measured with FC. (TIF) [file pone.0159515.s007.tif]
