# Supplementary material for: Fractionated Radiotherapy with 3 x 8 Gy Induces Systemic Anti-Tumour Responses and Abscopal Tumour Inhibition without Modulating the Humoral Anti-Tumour Response
Source: PLoS One. 2016 Jul 18;11(7):e0159515. doi: 10.1371/journal.pone.0159515 (PMC4948777; doi:10.1371/journal.pone.0159515)
Supplement: S1 File — Development of cellular read-out systems for the detection of TBAs in the plasma of Balb/C mice. (PDF) [file pone.0159515.s008.pdf]

S1 File. Supplementary Materials and Methods.

Development of cellular read-out systems for the detection of TBAs in the plasma of Balb/C mice.

In order to have a system that allows the detection of tumour-specific antibodies we set out to develop cellular-based detection assays using flow cytometry and ELISA. In order to establish whether these assays quantitatively reflect the antibody concentration or whether they are influenced by undesired protein factors in the plasma sample also defined as matrix effects we performed dilution experiments of an antibody of known concentration in different concentrations of plasma (S2b Fig). For this assay we stained 10^5 tumour cells with five concentrations (0.5, 0.1, 0.05, 0.01, 0.005 µg/µL) of a monoclonal unconjugated antibody against the MHC molecules (H-2K^d) of the cell line in 5 different concentrations of mouse plasma (1:2, 1:10, 1:20, 1:40, 1:80) or in buffer.

As shown in S2a Fig (right panel), the H-2K^d binding to the cells was dependent on the plasma dilution. In particular, a plasma dilution of 1:2 showed reduced binding of the H-2K^d recognizing antibody to the 67NR tumour cells. Additionally, in this plasma dilution no dose-dependency could be observed when more of the monoclonal antibody was added. These matrix effects were only observed in the 1:2 dilution of the plasma and all other plasma dilutions demonstrated a dose-dependent increase in antibody binding. Notably, the MFI values were highest in the condition where the plasma was diluted 1:10 and 1:20 and these MFIs exceed the MFI values observed in a typical buffer used for flow cytometry (PBS + 10% FCS + 0.05% Tween 20) with a lower protein content. With exceeding dilution of the plasma the MFI values resemble more closely those that are observed in buffer. Based on these data the highest signal to noise ratio was observed at dilutions above 1:10 and a plasma dilution of 1:20 was used for all antibody detection experiments. Using this serum

concentration there is a dose-dependent correlation between the MFI and the quantity of the antibody that is spiked in the plasma.

In order to evaluate whether this flow cytometric methodology also allows the detection of a *de novo* induced polyclonal antibody response in an animal, we vaccinated healthy Balb/C mice with 10^7 heat killed 67NR cells in incomplete Freund's adjuvant and CpG. Besides the generation of positive control sera, the secondary aim of this experiment was to study whether it is possible to generate antibodies against the 67NR tumour cell line in animals with the same genetic background as the cell line. Additionally, this vaccination experiment allows studying to what extent the induction of an antibody response against the tumour (after vaccination) is transient (and whether a booster vaccine was required for a durable antibody response). To this end we vaccinated animals with a cocktail of IFA, CpG ODN, and heat-killed 67NR cells to boost 67NR specific antibody responses. At week 3, 4, and 5 plasma samples were collected. Additional animals received a booster vaccine at week 3 and plasma was collected on week 1, 2, and 3 after the booster. In order to demonstrate the induction of an antibody response against 67NR we incubated 10^5 67NR cells with 1:20 diluted plasma of control and vaccinated mice. As shown in S3 Fig, we demonstrated antibody binding in the plasma collected 3 weeks after the first vaccination. However, this antibody response was not a durable response as antibody binding decreased in the plasma collected after 4 or 5 weeks. Upon boost vaccination we observed increased antibody binding to the 67NR cells that lasted until at least 3 weeks after the first vaccination. The sera that were collected after the boost vaccination were used to further optimize the flow cytometric and cellular ELISA-based methods.

Because both the sensitivity as the variability of antibody detection is dependent on the amount of antigen we performed titration experiments in which various numbers of 67NR tumour cells were incubated with 1:20 diluted plasma derived from healthy (-control) or vaccinated (S3 Fig) Balb/C mice. As shown in S2b Fig, the maximal signal to noise ratio

(vaccination to -control ratio) with minimal technical variation was reached when the experiment was performed with 1 $*10^5$ 67NR tumour cells per condition. The variation was increased in conditions where 75 $*10^3$ cells were used and the sensitivity decreased when more than 2 $*10^5$ 67NR tumour cells were used (S2b Fig).

In addition to the development of a flow cytometric detection system we also set out to develop an ELISA-based system for the detection of humoral antibodies. To this end we seeded tissue culture plates with different numbers of 67NR cells and incubated them for 18 hours. Although, 67NR cells showed a strong adherent phenotype (fibroblast-like structure), they detached from the culture plates during these washing steps. Therefore, we fixated 67NR cells with formaldehyde for 15, 20, 30, and 45 min. These results (not shown) indicated that 15 minutes was not sufficient, whereas 45 minutes was.

After washing the 67NR cells, we fixated the cells using paraformaldehyde for 45 minutes and blocked remaining binding sites with 1% BSA. After incubation with plasma of the animals we detected primary antibody binding using an HRP-labelled anti-mouse Ig. In order to validate this cellular ELISA-based protocol, we spiked plasma with the primary H-2K^d antibody as described above, followed by titration of the detection antibody with plasma from vaccinated and healthy Balb/C mice (S4a Fig). Using this assay, we demonstrated that seeding of 10^5 cells resulted in a higher signal and less internal variation as compared to seeding of 0.75 $*10^5$ cells. In addition, we questioned in these experiments whether the fixation protocol could account for background staining. Therefore, we determined the binding of the H-2K^d antibody and the secondary antibody Ig-HRP (background) on fixated 67NR cells (15, 20, 30 and 45 min). We observed no increased binding of the H-2K^d antibody with longer fixation protocols and even observed less binding of the secondary antibody with longer fixations (data not shown). Subsequently, we determined the optimal dilution for the plasma. Therefore, binding of antibodies of the vaccinated Balb/C mice and control mice was evaluated when plasmas were diluted 20, 40, 80, 160, and 320 times (S4b Fig). As

shown in S4b Fig, the largest percentual difference between vaccinated and negative control plasma was observed at a plasma dilution of 1:20.

In order to measure the Ig isotype composition in plasma of tumour-bearing mice we selected antibodies that show minimal crossreactivity with Igs from another subclass. Freshly isolated human peripheral blood mononuclear cells (PBMCs) were incubated with unconjugated mouse anti-human antibodies specific for CD28 (IgG_1), CD3 (IgG_{2a}), CD158b (IgG_{2b}), and CD158a (IgM) (S5a Fig). Binding of these antibodies was detected with an antibody cocktail containing rat anti-mouse IgG_1 (APC), IgG_{2a} (PE), IgG_{2b} (FITC), and IgM (PE-Cy7). As shown in S5b Fig, the gating on IgG_1 positive cells displayed no crossreactivity with the other subclass specific detection antibodies, which was also confirmed for the other subclasses. In all cases less than 5% of the MFI was observed when the mouse anti-human antibodies were stained with antibodies that are reactive with another subclass (S5c Fig).
